# 1220. Is MIC all that matters? MIC Distributions of Ceftazidime and Cefepime in Ceftriaxone-Resistant *E. coli* and *Klebsiella* spp

**DOI:** 10.1093/ofid/ofab466.1412

**Published:** 2021-12-04

**Authors:** Emily Heil, Emily Heil, Kimberly C Claeys, Paul Luethy

**Affiliations:** 1 University of Maryland School of Pharmacy; University of Maryland Medical Center, Baltimore, MD; 2 University of Maryland School of Pharmacy, Baltimore, Maryland; 3 University of Maryland School of Medicine, Baltimore, Maryland

## Abstract

**Background:**

The Clinical and Laboratory Standards Institute (CLSI) lowered MIC breakpoints for many beta-lactam antibiotics to enhance detection of resistance among *Enterobacterales*. This shift was also meant to eliminate the need for routine testing for extended-spectrum beta-lactamases (ESBLs). The recommended treatment for ESBL-producing *Enterobacterales* is carbapenems. The IDSA guidelines for MDR-GN organisms recommend using ceftriaxone (CRO) resistance as a proxy for ESBL production and thus carbapenem treatment. Under CLSI guidelines, alternative beta-lactams such as ceftazidime (CAZ) and cefepime (FEP) may still be reported as susceptible and thus used by clinicians even in light of IDSA recommendations. The aim of this project was to characterize the MIC distributions of CAZ and FEP stratified by CRO susceptibility.

**Methods:**

Clinical *E. coli*, *K. pneumoniae*, and *K. oxytoca* isolates from blood cultures in adult patients from Nov 2016-Dec 2018 that had MICs tested by the Vitek-2 automated susceptibility testing system for CRO, FEP and CAZ were identified. Descriptive statistics were used to compare MIC distributions across the antibiotics of interest (SPSS).

**Results:**

573 isolates were included, of these, 17.3% were CRO resistant. Most (53%) CRO-R isolates had FEP MICs ≤2 which is considered susceptible per CLSI; 19% had FEP MICs of 4-8 which would be considered S-DD by CLSI (Figure 1A; breakpoints noted by dashed lines). Using the EUCAST breakpoint of ≤1, only 11% of CRO-R isolates would be reported as FEP-S. For CAZ, 40% of CRO-R isolates had CAZ MICs ≤4, which is considered S by CLSI. Using the EUCAST breakpoint of ≤1, only 12% of CRO-R isolates would be reported as CAZ-S (Figure 1B).

Cefepime MIC Distribution for Ceftriaxone Resistant Isolates

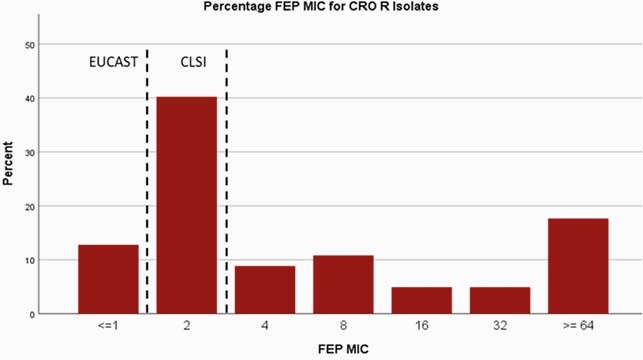

Distribution of MICs for cefepime for ceftriaxone resistant isolates with the breakpoints for EUCAST and CLSI noted with a dashed line

Ceftazidime MIC Distribution for Ceftriaxone Resistant Isolates

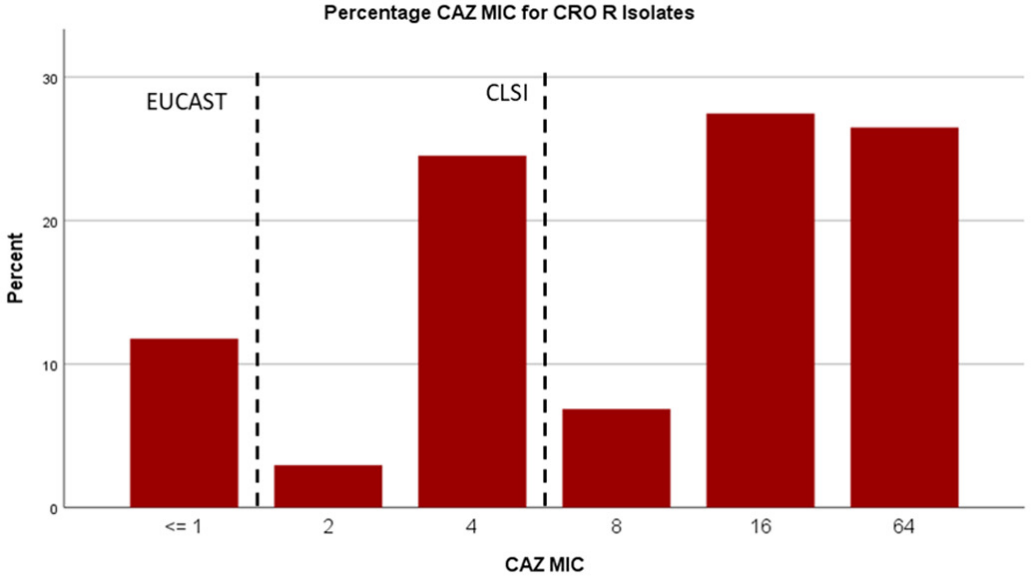

Distribution of MICs for ceftazidime for ceftriaxone resistant isolates with the breakpoints for EUCAST and CLSI noted with a dashed line

**Conclusion:**

Half of CRO-R *E. coli, K. pneumoniae* and *K. oxytoca* have FEP and CAZ MICs at or below the current CLSI breakpoints. This may lead to their use for serious ESBL infections where a carbapenem is preferred. To prevent unnecessary use, laboratories should consider suppressing FEP and CAZ susceptibilities when CRO-R or adopting more the aggressive EUCAST breakpoints for these agents.

**Disclosures:**

**Emily Heil, PharmD, MS, BCIDP**, Nothing to disclose **Kimberly C. Claeys, PharmD**, **GenMark** (Speaker’s Bureau)

